# From Diagnosis to Therapy in Primary Cutaneous Extramammary Paget’s Disease: A Systematic Review of Non-Invasive and Non-Surgical Approaches

**DOI:** 10.3390/cancers17213594

**Published:** 2025-11-06

**Authors:** Francesco D’Oria, Francesco Piscazzi, Matteo Liberi, Giulio Foggi, Luigi Lorini, Katia Maria Calcara, Emi Dika, Mario Valenti, Salvador González, Marco Ardigò

**Affiliations:** 1 Dermatology Unit, IRCCS Humanitas Research Hospital, 20089 Rozzano, Italy; francesco.doria@humanitas.it (F.D.); francesco.piscazzi@humanitas.it (F.P.); matteo.liberi@humanitas.it (M.L.); giulio.foggi@humanitas.it (G.F.); mario.valenti@hunimed.eu (M.V.); 2 Department of Biomedical Sciences, Humanitas University, Pieve Emanuele, 20072 Milan, Italy; 3 Medical Oncology and Hematology Unit, IRCCS Humanitas Cancer Centre, 20089 Rozzano, Italy; luigi.lorini@humanitas.it (L.L.); katia.calcara@cancercenter.humanitas.it (K.M.C.); 4 Department of Precision Medicine in Medical, Surgical and Critical Care (Me.Pre.C.C.), University of Palermo, 90133 Palermo, Italy; 5 Oncologic Dermatology Unit, IRCCS Azienda Ospedaliero-Universitaria di Bologna, 40138 Bologna, Italy; emi.dika3@unibo.it; 6 Department of Medical and Surgical Science, University of Bologna, 40126 Bologna, Italy; 7 Dermatology Service, Memorial Sloan-Kettering Cancer Center, New York, NY 10065, USA; salvagonrod@gmail.com; 8 Dermatology Service, Ramon y Cajal Hospital, Alcalá University, 28801 Madrid, Spain

**Keywords:** extramammary Paget’s disease, non-invasive diagnostics, reflectance confocal microscopy, photodynamic diagnosis, imiquimod, photodynamic therapy, non-surgical treatment, rare cutaneous malignancy

## Abstract

**Simple Summary:**

Extramammary Paget’s disease (EMPD) is a rare skin cancer that primarily affects the genital and perianal regions. Surgery remains the primary treatment option, but it often involves invasive procedures that can cause discomfort and functional impairment, and still carry a risk of recurrence. These limitations have stimulated interest in less invasive options. This review focuses on two main aspects. First, the role of imaging in detecting EMPD, defining lesion margins, and monitoring patients over time without repeated biopsies. Second, microscopic evidence is presented for non-surgical treatments such as topical agents, radiotherapy, or photodynamic therapy. By critically analyzing the literature, we explore how these approaches may help individuals who cannot undergo surgery or wish to avoid it. Our findings provide insights for future research and support more tailored management of patients with EMPD.

**Abstract:**

**Background/Objectives**: Extramammary Paget’s disease (EMPD) is a rare cutaneous malignancy arising in areas rich in apocrine glands that poses diagnostic and therapeutic difficulties. Although surgery remains the standard of care, achieving clear margins is challenging and recurrence rates are high. This review explores the contribution of non-invasive imaging for diagnosis and monitoring, and evaluates conservative, non-surgical therapies as alternatives to radical surgery. **Methods**: Following Preferred Reporting Items for Systematic Reviews and Meta-Analyses (PRISMA), a systematic review was conducted: eligible studies included interventional and observational research, as well as case series and reports, assessing non-invasive diagnostic methods or non-surgical treatments for EMPD. Data extraction and risk-of-bias evaluation were performed independently by multiple reviewers, and a narrative synthesis summarized therapeutic outcomes and diagnostic performance. **Results**: Of 808 identified records, 82 met the inclusion criteria: 66 focused on non-surgical therapies, 15 on diagnostic techniques, and one on both. Reflectance confocal microscopy (RCM) and photodynamic diagnosis (PDD) showed high concordance with histopathology, aiding both diagnosis and margin delineation. Among therapies, topical imiquimod and photodynamic therapy (PDT) demonstrated encouraging response rates, while radiotherapy, laser ablation, and systemic chemotherapy were less consistently reported. Evidence quality was limited by small cohorts, heterogeneous regimens, and variable follow-up. **Conclusions**: Non-invasive imaging enhances diagnostic accuracy and surgical planning, while non-surgical treatments—particularly imiquimod and PDT—offer viable alternatives in selected cases. Larger prospective studies are needed to establish standardized protocols and clarify long-term outcomes.

## 1. Introduction

Extramammary Paget’s disease (EMPD) represents an uncommon form of cutaneous malignancy that arises in areas rich in apocrine glands [1]. Its occurrence is exceptionally rare, with incidence estimates ranging from 0.1 to 2.4 cases per million individuals per year [2,3]. The disease predominantly affects the genital region, with the vulva being the most frequent site in women, followed by the penoscrotal area in men; other sites include the perianal region and, less commonly, the axilla. Among reported cases, EMPD is most often diagnosed in Caucasian women, particularly those aged between 50 and 80 years [1,2,3,4].

EMPD is a rare cutaneous malignancy that may arise through two distinct pathways, each carrying different prognostic significance. Primary EMPD originates within the epidermis, most often presenting as an intraepithelial (in situ) neoplasm, though it may progress to an invasive form capable of regional or distant metastasis. In some cases, it is associated with an underlying adenocarcinoma of skin appendages or subcutaneous glands. Secondary EMPD, by contrast, reflects epidermal involvement by malignant cells derived from non-cutaneous adenocarcinomas—most commonly anorectal, urothelial, or other visceral primaries—via metastasis or epidermotropic colonization [4,5]. These associated tumors may occur simultaneously or develop at a different time point [6,7,8,9].

The definitive diagnosis of EMPD relies on biopsy and anatomopathological assessment. Microscopically, the epidermis shows the presence of Paget cells, which are large with abundant pale, finely granular cytoplasm and pleomorphic round nuclei. These cells are usually scattered individually but may also appear in small clusters [4]. Immunohistochemical staining typically reveals positivity for cytokeratins, epithelial membrane antigen, and carcinoembryonic antigen [5].

Surgery remains the mainstay of care, most commonly performed as wide local excision (WLE) or Mohs micrographic surgery (MMS) [10]. The real challenge, however, lies in defining the true extent of the disease. EMPD may spread microscopically beyond what is visible, arise in multiple foci, and show irregular borders. As a result, obtaining histologically negative margins without resorting to extensive or even mutilating procedures is difficult. Recurrence is therefore common, emphasizing the importance of accurate margin assessment in shaping both prognosis and long-term outcomes [6,7,8,11,12].

Non-invasive imaging technologies have opened new possibilities for diagnosing and managing cutaneous malignancies. Techniques such as dermoscopy, reflectance confocal microscopy (RCM), photodynamic diagnosis (PDD), optical coherence tomography (OCT), positron emission tomography/computed tomography (PET/TC), and magnetic resonance imaging (MRI) enable visualization of skin architecture at various depths without the need for biopsy. Among these, RCM provides immediate feedback during surgery, making it especially valuable for delineating tumor margins and identifying residual disease. This real-time information can significantly improve the precision, speed, and overall outcomes of MMS and other operative strategies in EMPD treatment [5].

Over the years, several non-surgical approaches have been tried in EMPD, but their use is still quite limited. This is partly because the disease is rare, and partly because we do not yet have large, controlled studies to guide practice [13,14,15,16]. Reported alternatives include radiotherapy, photodynamic therapy (PDT), laser treatment, and different topical agents. Among these agents, imiquimod has attracted particular interest, showing favorable outcomes in certain clinical contexts [17,18,19,20,21,22]. This drug, a toll-like receptor 7 agonist, works by stimulating the innate immune system and triggering cytokines such as interferon-α, interleukin-6, and Tumor Necrosis Factor α (TNF-α), which together contribute to an antitumor effect. Although approved for actinic keratosis, superficial basal cell carcinoma, and genital warts, clinicians have extended its use to EMPD [4]. Other topical creams, like 5-fluorouracil, have also been tested, while systemic chemotherapy is generally considered only when the disease is invasive or metastatic [5].

When limited to the epidermis and managed appropriately, primary EMPD is usually associated with a good prognosis. In contrast, once the disease becomes invasive and involves regional lymph nodes or distant organs, systemic therapy is often required and the outlook is considerably poorer [22].

This systematic review focuses on non-invasive and conservative strategies for the management of EMPD, integrating diagnostic and therapeutic perspectives within a unified framework. Non-invasive imaging techniques are central, as they improve the accuracy of disease detection, margin assessment, and postoperative surveillance, thereby informing and optimizing treatment decisions. In parallel, conservative non-surgical therapies are discussed as complementary options that align with the same minimally invasive philosophy—offering meaningful clinical benefit for patients in whom radical surgery is contraindicated or undesirable. Together, these approaches represent, for the first time, a comprehensive continuum of care that unites diagnosis and treatment within a single patient-centered management strategy, emphasizing the evolving paradigm toward less invasive yet effective disease control.

## 2. Materials and Methods

### 2.1. Study Design

**Figure 1:**
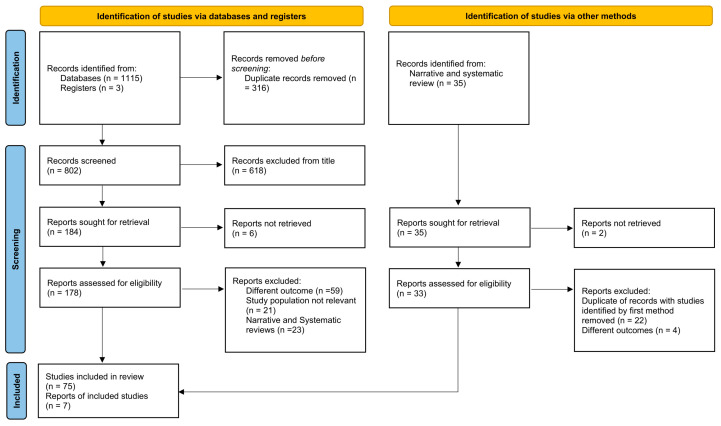
PRISMA flow chart illustrating the study selection process of studies. The chart shows records identified through databases, registries, and other sources, followed by screening, eligibility assessment, and final inclusion in the review. A total of 82 studies (75 from articles and 7 articles retrieved from narrative and systematic review) were included after removing duplicates and excluding records due to irrelevance or ineligible outcomes.

This systematic review was conducted in compliance with the Preferred Reporting Items for Systematic Reviews and Meta-Analyses (PRISMA) guidelines (Figure 1) [23].

### 2.2. Inclusion and Exclusion Criteria

Given the scarcity of research on this subject, we adopted broad eligibility criteria. We considered all interventional studies, whether randomized or not, carried out in male or female patients of any age and from any geographical area. To be eligible, studies had to provide data on healing rates at different time points, outcomes of non-surgical therapies, local recurrence rates, and management or follow-up using non-invasive imaging methods. Studies focusing on patients with EMPD secondary to an underlying malignancy or in advanced stages of disease were excluded.

We also accepted conference abstracts and proceedings, as well as single case reports, since these often represent valuable individual experiences that would otherwise remain underrepresented in the literature. Conversely, narrative reviews, systematic reviews, and meta-analyses were initially set aside. During the data extraction phase, articles referenced within these reviews were assessed for eligibility, and those meeting the inclusion criteria, without duplication of previously included studies, were extracted and included in the analysis (See Section 2.4). Finally, only articles written in English, Spanish, French, German, or Italian were included in the review.

### 2.3. Search Strategy

A comprehensive literature search was conducted to identify studies evaluating non-surgical therapies and non-invasive diagnostic techniques for EMPD. Searches were performed up to July 2024 across five major databases: PubMed, Ovid Embase, Cochrane CENTRAL, Web of Science Core Collection, and ClinicalTrials.gov. Two independent reviewers (F.P. and M.V.) performed the search and screening processes, with a third reviewer (M.A.) resolving disagreements. No restrictions were applied regarding publication date, language, or study design. The search strategy was developed around four core concepts following Patient/Population, Interventions, Comparison, and Outcome strategy. Patient/Population: EMPD; Interventions: non-surgical and topical treatments like imiquimod, fluorouracil, PDT, radiotherapy and non-invasive diagnostic tools like RCM and PDD; Comparison: not applicable; Outcome terms like recurrence, diagnosis, follow-up, retreatment. All retrieved records were exported to Endnote for automatic de-duplication and then imported into an excel file for independent screening of titles/abstracts and full texts. When abstracts were later published as full articles, the full articles were given priority over the abstracts.

### 2.4. Data Extraction

During the initial screening phase, all records retrieved from the database were reviewed by two independent investigators (M.V. and F.P.) and a third reviewer (M.A.) in case of disagreement. From an initial set of 802 references, 618 were excluded after evaluation of titles only. The remaining studies underwent a second-level screening, which required careful examination of abstracts, and, when necessary, consultation of the results section, aimed at verifying whether the outcomes of interest were reported. Specifically, we considered: (i) healing rates at 6 months, 1 year, and 3 years for non-surgical treatments (including imiquimod, radiotherapy, photodynamic therapy, 5-fluorouracil, or other therapies); (ii) local recurrence rates at different time points (to be defined in the synthesis stage); and (iii) the use and role of confocal microscopy in the follow-up of EMPD.

The intermediate screening of abstracts was conducted independently by two authors (F.D’O. and M.L.). Each study was coded in a predesigned Excel file: articles judged relevant by both reviewers were assigned a score of “1,” while non-eligible studies were assigned “0.” In cases of disagreement between the two reviewers, a third author (F.P.) acted as an adjudicator. After this step, 75 studies were retained for study inclusion. A total of 35 articles were retrieved from 23 narrative and systematic reviews with titles consistent with the outcomes of interest by two authors (F.D’O. and M.L.). In cases of disagreement between the two reviewers, a third author (F.P.) acted as an adjudicator. Of these, only 7 were ultimately included in the final analysis, as most were duplicates of studies already identified through the primary search strategy, while a smaller proportion were excluded due to reports not being retrievable or because they addressed different outcomes. In total, 82 studies were included in the final review.

### 2.5. Risk of Bias

The methodological quality of the selected studies was independently assessed by two reviewers (L.L. and K.M.C.) in accordance with the Risk Of Bias In Non-randomized Studies-of Interventions (ROBINS-I) tool, applying the most appropriate domains for each study. The evaluation considered potential confounding, selection of participants, classification and deviations from intended interventions, missing data, measurement of outcomes, and selection of reported results. Two reviewers independently assessed each study (L.L. and K.M.C.), and disagreements were resolved through discussion. When necessary, consensus was reached with the involvement of a third author (F.P.). The assessment of methodological rigor and potential sources of bias was incorporated into the overall interpretation of the findings.

### 2.6. Statistical Analysis

A narrative synthesis was carried out, supported by summary tables that combined therapeutic and diagnostic information from the included studies. The analysis encompassed treatment characteristics (such as prior management, schedules, outcomes, recurrence and adverse effects) together with data on non-invasive diagnostic strategies, their aims and main findings. Continuous variables were summarized using means or medians. Categorical variables were analyzed and presented as frequencies and percentages. Due to the heterogeneity and limited availability of data, no additional analyses were conducted. When information was incomplete, calculations were based on the number of cases or studies for which data were available.

## 3. Results

### 3.1. Study Selection

The PRISMA flow chart (Figure 1) illustrates the process of study selection. Ultimately, 82 studies were retained for qualitative synthesis: 66 addressing non-surgical therapeutic strategies for EMPD, 15 focusing on non-invasive diagnostic approaches, and one study contributing to both categories.

### 3.2. Study Quality, Bias Results and Limitations

Overall study quality was heterogeneous. Most publications were case reports or small case series, while observational designs were less frequent. In the therapeutic literature, the absence of control arms was common, and outcome assessment was usually descriptive, with limited use of standardized measures or formal statistical analysis. Reporting of recurrence, retreatment, and adverse events was inconsistent, and follow-up durations varied widely across studies. Diagnostic investigations were similarly limited, often consisting of feasibility assessments in small cohorts without systematic comparisons to reference standards. Methodological details such as patient selection, timing of assessment, and interpretation criteria were frequently incomplete. Taken together, the evidence base was characterized by variability in study design, reporting quality, and data completeness.

As most of the included studies were small case series rather than randomized controlled trials, this may have introduced a certain degree of bias and limited the robustness of the conclusions. A publication bias cannot be entirely excluded, as smaller reports are more likely to present positive findings. Furthermore, because conference abstracts and proceedings were included to ensure comprehensive coverage of the literature, some overlap with subsequently published full articles may have occurred, potentially leading to partial duplication of cases. Obvious duplicates were excluded whenever identified, and this limitation was considered in the interpretation of the findings.

The included studies were highly heterogeneous in terms of design, patient characteristics, disease stage, treatment modality, and outcome measures. Therefore, a statistical pooling of results was not methodologically appropriate, and findings were summarized descriptively. This heterogeneity, combined with the lack of controlled or comparative trials, restricts the generalizability of the conclusions and prevents firm recommendations regarding optimal diagnostic or therapeutic strategies.

In addition, the literature search was completed in July 2024, corresponding to the period of data extraction and analysis. Although no significant new studies were identified in a supplementary check performed before submission, the potential time lag between the last search and publication represents a limitation.

### 3.3. Study and Population Characteristics

A total of 82 studies met the inclusion criteria: 66 investigating therapeutic strategies, 15 focusing on diagnostic approaches, and one article contributing to both categories. Therapeutic reports described a wide range of non-surgical options, including topical agents such as imiquimod [24,25], PDT in different settings [24], radiotherapy either as a primary or adjuvant treatment [25], laser procedures, and less conventional strategies such as boron neutron capture therapy [26] or systemic chemotherapy [27]. Sample sizes varied substantially, from single-patient case reports to larger cohorts exceeding 90 individuals [28]. Diagnostic studies mainly assessed RCM and PDD, ranging from individual case descriptions [29] to broader series such as Tan et al., which included 73 patients [30], generally reporting concordance with biopsy findings, margin definition, and characteristic imaging features of Paget cells. Across all reports, details on lesion size, disease duration, and number of lesions were often missing, and follow-up periods ranged from a few months to more than five years [31].

### 3.4. Diagnostic Characteristics

Sixteen studies evaluated non-invasive diagnostic techniques for EMPD (Table 1). The majority of these studies investigated the use of RCM, either alone or combined with PDD. Sample sizes ranged from single-patient case reports [29] to larger cohorts, such as that by Tan et al. with 73 patients [30] and Huang et al. with 36 patients and 130 sections [32].

RCM was evaluated in a single study for the primary diagnosis, detection of residual disease, and assessment of surgical margins in patients with recurrent EMPD. Of the 22 clinically suspicious sites examined in 5 patients (4 men, 1 woman; median age 70 years, range 56–77), 9 (40.9%) were positive for recurrent disease on high-resolution confocal microscopy and histopathologically confirmed, while 13 (59.1%) were negative on RCM, with 3 of these 13 positive for EMPD on histopathology. Overall, RCM demonstrated a sensitivity of 75% and a specificity of 100% in detecting recurrent or persistent EMPD. False-negative results occurred in two patients, mainly at lesion margins near previous biopsy sites. The use of video mosaicking appeared to improve lesion detection [33,34].

Commonly described features included large hyper-reflective Paget cells within a disrupted honeycomb pattern, glandular nests at the dermoepidermal junction, and “target cells” with a bright nucleus and a peripheral dark halo [32,35].

PDD, alone or in association with RCM, was mainly applied for margin delineation. Cheng et al. [35] reported that surgical margins required for clearance ranged between 0.5 and 2.0 cm beyond the visible lesion. Huang et al. [32] documented that 63.8% of tumor margins extended beyond the macroscopic boundary, while the combination of PDD with RCM reduced this proportion to 20.8%.

Follow-up data were reported inconsistently. Cheng et al. observed local recurrence in 6 of 36 patients (15.4%) within 12 months, and one case of nodal metastasis at 36 months [35]. Zhou et al. [36] described recurrence rates of 26.7% and 28.6% in control groups, compared with 4% in the experimental group using Wood lamp plus 5-aminolevulinic acid-mediated photodynamic therapy (5-ALA-PDT). Several single-patient reports documented absence of relapse during follow-up periods of up to two years [35,37].

**Table 1:** Summary of studies on non-invasive diagnostic techniques in EMPD, including sample size, type and purpose of the method (diagnosis, residual disease, or margin detection), timing, concordance with histopathology, key RCM features, treatment context, recurrence, and follow-up.

Authors	No. of Patients and Sections	Type of Non-Invasive Diagnosis	Purpose (Diagnosis, Monitoring, Margin Detection)	Time (Before or After Treatment)	Concordance to Different Approaches	RCM Features of Lesions	Type of Treatment	Recurrence	Follow-Up Period (Years)
Zhou et al. [36]	15 21	(A) Wide local excision (control group, 15 patients) (B) Wood lamp examination + 5-ALA-PDT (experimental group, 21 patients)	Margin detections	Pre-treatment	-	-	Surgery	Recurrence rate (A) 26.7% (B) 28.6%	4.0
Zhan-Yan et al. [38]	14 23	Reflectance confocal microscopy	Diagnosis and margin detections	-	Biopsy confirmed	Typical Paget cells were characterized by a mild bright nucleus and dark cytoplasm, frequently twice the size of keratinocytes or larger. At the dermoepidermal junction, tumor nests were seen as dark glandular structures	Surgery	-	-
Guitera et al. [39]	10 -	Reflectance confocal microscopy	Diagnosis	-	Biopsy confirmed	Presence of large atypical Pagetoid cells	Surgery	-	-
Debarbieux et al. [40]	1 -	Reflectance confocal microscopy	Diagnosis and margin detection	Pre-treatment and intraoperative	Biopsy confirmed	Intra-epidermal large dark isolated or nested cells, with few of them exhibiting a target appearance.	Surgery	-	-
Suppa et al. [31]	1 -	Reflectance confocal microscopy	Diagnosis	-	Biopsy confirmed	Large round cells with abundant and hypo-reflective cytoplasm with peripheral dark halo at epidermis.	Previously treated with topical 5% imiquimod cream	Recurrence after 13 years since first treatment	-
Terrier et al. [37]	1 -	Reflectance confocal microscopy combined with the “Spaghetti” Technique	Margin detection	Pre-treatment	Histological examination identified minimum clear margins of 3 mm	Dark and roundish cavities in the epidermis, corresponding to Paget cells.	Surgery	-	2.0
Yélamos et al. [41]	5 22	Reflectance confocal microscopy	Margin detection and residual disease	Pre-treatment	Three false-negative RCM at the margins of EMPD, close to previous biopsy sites.	Target cells with bright center and peripheral dark halo forming nests of Paget cells at DEJ. Focal dark holes in the stratum spinosum.	Surgery, topical 5% imiquimod cream, radiotherapy plus oral ERBB2-TKI	Recurrent disease on handheld RCM and histopathologically confirmed: 9/22 (40.9%); Negative on HRCM: 13/22 (59.1%), of which 3 were positive for EMPD on histopathological examination.	-
Chuchvara et al. [29]	1 -	Reflectance confocal microscopy	Diagnosis	-	Similarities to melanoma on dermoscopy, histopathology, and RCM versus immuno-histochemistry staining revealing pigmented EMPD	Atypical hyperreflective dendritic cells and hyporeflective round nucleated cells within a disarranged honeycomb pattern at the level of epidermis and DEJ	-	-	-
Wu et al. [32]	36 130	Photodynamic diagnosis plus reflectance confocal microscopy	Margin detection	Pre- and post-treatment	Tumor margins beyond macroscopic line: 83/130 (63.8%) Tumor margins beyond photodynamic diagnosis marker line: 46/130 (35.4%) Tumor margins beyond photodynamic diagnosis plus reflectance confocal microscopy marker line: 27/130 (20.8%)	Highly refractive, large-nucleated cells on the epidermis	Surgery	-	-
Kibbi et al. [42]	33 41	Reflectance confocal microscopy	Margin detection	Post-treatment	RCM correlation with scouting punch biopsies (kappa, 0.93)	Dark holes in the epidermis (Paget’s cells) and glandular nests of cells	Surgery: 21/33 Radiotherapy: 5/33 Imiquimod 6/33 Photodynamic therapy: 1/33	-	-
Huang et al. [34]	1 -	Photodynamic diagnosis plus reflectance confocal microscopy	Diagnosis and margin detection	Pre-treatment	-	Highly refractive, large-nucleated cells on the epidermis	Surgery	-	-
Ganhewa et al. [43]	1 -	Reflectance confocal microscopy	Residual disease	Pre-treatment	-	Paget cells with distinctive holes in the honeycomb’ pattern of keratinocytes	5-FU	-	-
Navarrete-Dechent et al. [44]	33 36	Reflectance confocal microscopy	Margin detection	Post-treatment (mean margin needed to clear 1.8 cm)	RCM correlation with scouting punch biopsies (kappa, 0.93; *p* < 0.001)	-	Surgery	-	1.5
Tan et al. [30]	73 -	Reflectance confocal microscopy	Diagnosis	-	RCM: 54/73 (74.0%) Biopsy: 52/67 (77.6%)	Disarranged honeycomb pattern in the upper epidermis and basal lamina; Paget cells in stratum spinosum; inflammatory cells in dermis; dilated vessels in tortuous morphology in the superficial dermis.	-	-	-
Filonenko et al. [45]	1 -	Photodynamic diagnosis	Margin detection	Before first and second cycle of treatment	-	-	Photodynamic therapy	Relapse-free follow-up at 2 years and 3 months after treatment	2.3
Cheng et al. [35]	36 166	Photodynamic diagnosis plus reflectance confocal microscopy	Margin detection	Post-treatment (mean margin needed to clear 0.5–2.0 cm)	-	-	Surgery	Local recurrence: 6/36, 15.4% (2–12 months postoperatively) Lymph node metastasis without local recurrence: 1/36, 2.8% (36 months postoperatively)	3.0

### 3.5. Treatment Characteristics

A total of 67 studies reported non-surgical therapies for EMPD (Table 2). Among them, topical imiquimod 5% was the most frequently investigated. When considering only monotherapy cohorts with clearly reported outcomes, data extracted from Hata et al., Clément et al., and Rioli et al. indicated a complete response (CR) in 57.9% of patients (133/230). Partial responses (PR) were seen in 31.0% (38/124), and stable disease (SD) in 9.5% (2/21) of cases. Disease progression was rarely documented [25,46,47].

Photodynamic therapy was the second most commonly evaluated modality. Across the available studies evaluating PDT as monotherapy, the CR rate was 83.1% (83/100) [26,48]. PR was reported in 24.8% (6/24), SD in 42.4% (6/14), and PD in 7.0% (1/13) of patients [49].

Radiotherapy was described in multiple case series, often after surgery, but also as primary treatment, with generally favorable outcomes [27,43].

Other approaches were less frequently reported, including laser ablation [49], topical 5-fluorouracil [43], systemic chemotherapy [27], and boron neutron capture therapy [26].

Treatment regimens were highly heterogeneous. Imiquimod was applied between 2 and 7 times weekly, while PDT protocols varied in photosensitizer, light source, and number of cycles. Follow-up ranged from a few months in case reports to over five years in larger series [50].

**Table 2:** Summary of studies on non-surgical therapeutic approaches for EMPD, including patient numbers, type and line of treatment, previous therapies, treatment regimens, clinical outcomes (CR, PR, SD, PD), recurrence and retreatment data, reported adverse effects, and follow-up duration.

Authors	No. of Patients	Type of Non-Surgical Treatment	Line of Treatment	Previous Treatments	Dosage and Time	Therapeutic Outcome CR, PR, SD, PD (Months)	Recurrence: Yes/No (Months)	Retreatments	Side Effects	Follow-Up Period (Years)
Escolà et al. [51]	76	Topical 5% imiquimod cream Photodynamic therapy (ALA in 20.5% and mALA in 79.5%) Topical 5-Fluorouracil Radiotherapy (82.4% external beam radiotherapy with photons or electrons, 11.8% brachytherapy and 5.9% orthovoltage radiotherapy) Carbon dioxide laser	First line	-	Dose schedules ranged from once daily to twice weekly and duration from 1 to 104 weeks Total of 1–10 treatments at intervals ranging from 1 to 5 weeks Once or twice daily for 2–10 weeks Doses ranged between 8 and 64 Gy delivered in 1–33 fractions -	CR 52.2%; PR 30.6%; SD 13.4%; PD 3.7% (<3 months) CR 14.6%; PR 45.8%; SD 33.3%; PD 6.3% (<3 months) CR 18.8%; PR 37.5%; SD 37.5%; PD 6.3% (<3 months) CR 65.2%; PR 26.1%; SD 4.4%; PD 4.4% (<3 months) CR 0.0%; PR 50.0%; SD 25.0%; PD 25.0% (<3 months)	-	-	-	5.0
Xiang et al. [52]	1	Hematoporphyrin injection photodynamic therapy	First line	-	Intravenous dose of Hematoporphyrin Injection (HiPorfin; 5 mL: 25 mg) at 5 mg/kg in 250 mL saline. Laser wavelength 630 nm, power density 100 mW/cm^2^, irradiation duration 30 min per light spot, energy density 180 J/cm^2^	CR 100% (41 months)	No (41 months)	No	Redness, necrosis, scab, ulceration, granulation, scarring	3.4
Filonenko et al. [45]	1	Chlorin e6 photodynamic therapy	First line	-	Chlorin e6 intravenous 1.0 mg/kg. Irradiation (λ 662 nm, power density 130–150 mW/cm^2^, 194 min, light dose 300 J/cm^2^)	CR 100% (6 months)	Yes (21 months)	Photodynamic therapy (second cycle)	Redness, pain, bleeding, ulceration, scarring	4.0
Xiang et al. [53]	1	Hematoporphyrin photodynamic therapy	First line	-	Hematoporphyrin injection (HpD) 5 mg/kg intravenously in 250 mL saline solution. Laser wavelength 630 nm, power density 100 mW/cm^2^, irradiation time 30 min per spot, energy density 180 J/cm^2^	CR 100% (3 months)	Yes (18 months)	No	Pain, swelling, redness, scarring	3.5
Do et al. [54]	11	Radiation therapy	Adjuvant	Surgical treatment	-	-	-	-	-	2.9
Borella et al. [50]	51	Topical 5% imiquimod cream	First line	-	2 or 3 applications weekly, from a minimum of 24 weeks to a maximum of 72 weeks	CR 43.1% (66 months)	-	-	Erosions, burning, fever	5.5
van der Linden et al. [55]	23	Topical 5% imiquimod cream	First line	-	3 times a week for 16 weeks	CR 52.2%; PR 30.4% (12 months)	Yes (31 months)	Topical 5% imiquimod cream Surgery	Fatigue, headache	2.6
Wang et al. [56]	11	Hematoporphyrin derivatives photodynamic therapy	First line	-	HpD injection 3 mg/kg or 5 mg/kg plus 250 mL normal saline in 60 min. Fluorescence at 48 and 72 h. Laser 630 nm red light, dosage level 150–200 J/cm^2^	CR 90.1%; PR 9.1% (1 month) CR 72.7% (17.4 months)	No	No	Pain, infection, photosensitivity and uroschesis	1.5
Sadko et al. [57]	1	Topical 5% imiquimod cream	Adjuvant	Surgical treatment (vulvectomy)	3 times a week for 1 month, suspended for 2 weeks and resumed for 2 months	CR 100% (10 months)	No	No	Erythema	0.8
Ferrara et al. [49]	10	Fractional carbon dioxide laser abrasion, followed by photodynamic therapy	First line	-	Fractional carbon dioxide laser abrasion. 3 h occlusive application of ALA, 100 J/cm^2^ irradiation, 630 nm lamp. Combination repeated every 2 weeks for a total of 5 times	CR 20%; PR 80% (12 months)	Yes (12 months)	No	Swelling, pain, residual hyperpigmentation	1.0
Ganhewa et al. [43]	1	Topical 5-fluorouracil	First line	-	-	PR 100% (6 months)	-	-	Erythema	0.5
Liu et al. [58]	119 (including surgical patients)	Topical therapy (imiquimod, 75%), radiation, chemotherapy, photodynamic therapy	First line and adjuvant	Surgical treatment	-	-	Yes	Surgical treatment (80%)	-	4.7
Preti et al. [21]	17	Topical 5% imiquimod cream (13) Laser vaporization (4)	First line	-	3–4 applications weekly -	-	-	-	-	7.9
Tanimura et al. [59]	6	Radiotherapy	First line	-	51 Gy	CR 100% (6 months)	Yes (13 months)	Surgical treatment	Erosions and candidiasis	2.7
Rioli et al. [47]	13	Photodynamic therapy	Second line	Laser, surgery, imiquimod, PDT, fluorouracil, radiotherapy, brachytherapy, cryotherapy, ingenol mebutate	Topical 16% methyl aminolevulinate (MAL); red light (630 nm) fluency 37 J/cm^2^ for 7 to 10 minutes	CR 15%; PR 38%; SD 38%; PD 7% (3 months)	Yes (5 months)	Topical 5% imiquimod cream, surgery, carbon dioxide laser, cryotherapy, ingenol mebutate, fluorouracil, radiotherapy	Pain	3.2
Bauman et al. [60]	1	Topical 5% imiquimod cream Photodynamic therapy	First line	-	Once for 5 days per week for 1 month, followed by 2 months for 3 nights a week. Monthly 5-ALA photodynamic therapy for 6 months. After 6 months, imiquimod discontinued and quarterly photodynamic therapy performed	CR 100% (12 months)	No	-	-	5.0
Nitecki et al. [61]	20	Topical 5% imiquimod cream	Adjuvant (15) First line (5)	Surgical treatment	-	-	Yes (12.5 months)	Topical 5% imiquimod cream	Local irritation	3.8
Sawada et al. [62]	9	Topical 5% imiquimod cream	First line	-	3 times per week for 16 weeks; one case for 6 weeks	CR 56%; PR 44% (4 months)	Yes (34 months)	Topical 5% imiquimod cream	Local irritation	3.4
van der Linden et al. [7]	24	Topical 5% imiquimod cream	First line	-	3 times a week for 16 weeks	CR 52.2%; PR 30.4% (7 months)	Yes (12 months)	Topical 5% imiquimod cream Surgery	Fatigue and headache	2.6
Vicentini et al. [63]	1	Unconventional PDT	Next line	Imiquimod applications, LASER treatments and conventional photodynamic therapy	3 PDT sessions with 16% methyl-aminolevulinate cream with the light emitting fabric at irradiance 6 mW/cm^2^, fluence. 37 J/cm^2^	SD 100% (2 and 5 months)	-	-	-	0.4
Dogan et al. [64]	1	Topical 5% imiquimod cream	Adjuvant	Surgical resection and re-resection	Twice weekly was applied for 3 months	CR 100% (3 months)	No	-	Erythema	0.5
Knight et al. [65]	1	Topical 5% imiquimod cream	First line	-	Once for 5 days and 2 rest days per week for 4 months	CR 100% (4 months)	Yes (18 months)	-	Viral infection	1.5
Cowan et al. [66]	8	Topical 5% imiquimod cream	Second line	Surgical treatment	3 times per week for 3 months	CR 75%; PR 13% (3 months)	Yes (35 months)	Topical 5% imiquimod cream	Erythema	2.9
Higgins et al. [67]	1	Topical 5% imiquimod cream	Adjuvant	Surgical treatment	3-times weekly for 19 months	PR 100% (3 months); CR 100% (9 months)	Yes (9 months)	Topical 5% imiquimod cream	-	4.0
Al Youssef et al. [68]	1	Photodynamic therapy	First line	-	Topical methyl 5-aminolevulinate (5-MAL) and exposure to 37 J/ cm^2^ of visible red light (630 nm). Three PDT sessions at 4-week intervals	CR 100% (3 months)	No	-	Pain	1.0
Gao et al. [24]	38	Photodynamic therapy	Adjuvant (31) First line (7)	Surgical treatment	Aminolevulinic acid (5-ALA) and exposure to 120 J/cm^2^ with 635 nm laser for 15 min, for 3 times (adjuvant) or 4–6 times (first line)	CR 100% (<6 months)	Yes (6 and 12 months)	-	Pain and swelling	1.0
Marchitelli et al. [69]	10	Topical 5% imiquimod cream	Adjuvant (3) First line (7)	Surgical treatment	Every other day until the lesions were no longer clinically detected	CR 90%; PR 10% (5 months)	No	-	Irritation	1.5
Jing et al. [70]	2	Photodynamic therapy and topical 5% imiquimod cream combination	Next line	CO2 laser therapy Cryosurgery	6 cycles of 20% 5-aminolevulinic acid (ALA) photodynamic therapy and topical imiquimod	CR 100% (6 and 12 months)	No	-	-	3.0
Luyten et al. [71]	21	Topical 5% imiquimod cream	Adjuvant (6) First line (15)	Surgical treatment	2 or 3 times per week for a mean duration of 15.4 weeks	CR 52.4%; PR 28.6%; SD 9.5% (4 months)	No	-	Local reaction	-
Hata et al. [72]	41	Radiotherapy	Adjuvant (17) First line (24)	Surgical treatment	Total doses of 45–80.2 Gy (median, 60 Gy) were delivered in 23–43 fractions (median, 33 fractions). Irradiation took place 5 days per week and fraction sizes were 1.8–2.2 Gy (median, 1.8 Gy)	CR:100% (2–9 months)	Yes (3–44 months)	-	All patients had ≤grade 2 dermatitis, 13 ≤grade 2 colitis, 12 cystitis, 16 ≤grade 2 hematologic toxicities	3.4
Itonaga et al. [73]	14	Radiotherapy	Definitive (3) Definitive after relapse (6) Adjuvant (5)	Surgical treatment	Median total irradiation dose was 50 Gy, delivered in 20–33 fractions	CR 100% (71, 4 months)	Yes (13–83 months)	-	Local reaction	6.0
Fontanelli et al. [48]	32	Photodynamic therapy (M-ALA)	First line (32)	-	M-ALA PDT treatment was repeated every 3 weeks	CR 3.9%, PR 78.1%, NC 12.5% (11 months)	Yes (6, 10, 18 months)	Surgical treatment	-	1.5
Mann et al. [74]	1	Radiotherapy	First line	-	cumulative dose was 6400 cGy delivered in 200 cGy fractions over 6.5 weeks	CR	no	-	-	5.0
Clément et al. [46]	8	Photodynamic therapy	First line	-	3 h after topical application of methyl aminolevulinic acid emulsion, they underwent illumination with red light (570–670 nm) at a dose of 37 J/cm^2^ for 10 min. In the event of relapse, a further cycle was given at week 6.	CR 87.5% (3 months), PR 12.5%	Yes (4–14 months)	-	-	1.2
Hata et al. [25]	22	Radiotherapy	First line	-	A total dose of 45–70.2 Gy was delivered in 25–39 fractions (median, 33)	CR 86.3%, NC 13.7% (8–133 months)	Yes (3–43 months)	Surgical treatment	Local reactions	5
Green et al. [75]	27	Topical 5% imiquimod cream	First line	Surgical treatment	Once daily for 14 weeks	CR 78% (3 months)	Yes (11 months)	-	Lesional tenderness and erythema	1.0
Qiang et al. [76]	17	Photodynamic therapy	First line	-	Topical 20% 5-aminolevulinic acid was applied for 6 h. Each lesion was irradiated with 633 nm red light three times, 1 week apart, at a total dose of 339 J/cm^2^	CR 52.4% (6 months)	Yes (3 months)	-	Local reactions	2.0
Housel et al. [77]	8	Photodynamic therapy	First line	-	Four patients received topical ALA only as a photosensitizer, three received intravenous porfimer sodium only, and one received both. 632.8 nm argon-pumped dye laser, and some were also treated using a red lamp (590–729 nm)	-PDT using intravenous porfimer sodium CR 78% -PDT using topical ALA showed a CR 50% (9–88 MONTHS)	-	-	Local reactions	8.0
Fukui et al. [78]	5	Photodynamic therapy following carbon dioxide laser	-First line (2 patients) -neoadjuvant (3)	Surgical treatment	Carbon dioxide (CO2) laser abrasion, followed by 3 h of occlusive application of aminolaevulinic acid (ALA) and then 100 J/cm^2^ irradiation with a 630 nm excimer dye laser. This combination treatment regime was repeated every 2 weeks for a total of 3 times	CR 100 % (6 weeks)	Yes (12 months)	-	Local reactions	1.0
Tae Heung et al. [79]	1	Radiotherapy	Third line	Failure of local excision. Imiquimod 5% cream for two months	A total of 5040 cGy in 28 fractions was given	CR 100%	No	-	-	1.0
Geisler et Manahan [80]	1	Topical imiquimod 5% cream	Second line	Surgical treatment (multiple resections)	Once daily for 3 months	CR 100%	No	-	-	1.0
Vereecken et al. [81]	1	Topical imiquimod 5% cream	First line	-	Once daily for 3 months	CR 100%	No	-	Local erythema	1.0
Raspagliesi et al. [82]	7	Photodynamic therapy			5 MAL-PDT was applied for 3 h and than irradiated with red-light (620 nm) using a total light dose of 37 J/cm^2^ for a period of 10 min. Patients were treated once every 3 weeks, for a total of three treatments	CR 57% (4 months)	-	-	local edema and mild-moderate local pain	0.5
Holt et Stanley [83]	1	Radiotherapy	First line	-	40 Gray in 10 fractions	CR 100%	-	-	Mild local reactions	3.0
Madan et al. [84]	1	Photodynamic therapy	First line	-	ALA was applied followed 6 h later by irradiation using a filtered xenon-arc lamp	CR 100%	Yes (9 months)	intravenously administered porfimer sodium followed by one topical PDT treatment	-	1.5
Seok-Hyun et al. [85]	3	Radiotherapy	First line	-	54–78 Gy delivered in 6–8 weeks	CR 100%	Yes (2 years)	None	Local desquamation, mild late atrophic skin changes	0.5–8-11
Mikasa et al. [86]	2	Photodynamic therapy	First line	-	ALA-PDT treatments were applied to parts of the lesions at a total dose of 200–300 J/cm^2^	CR 100%	Yes (2 months)	two more PDT treatments	-	0.6
Luk et al. [87]	6	Radiotherapy	First line (2), postexcisional relapse (3) and adjuvant treatment (1)	Surgical excision	60 Gy delivered at 2 Gy/fraction, 5 fraction/week	CR 83.3%, PR 16.7%	Yes (18 months)	Surgical treatment	Acute confluent wet desquamation and mild late skin atrophy	1.2–14.8
Berman et al. [88]	1	Topical imiquimod 5% cream	First line	-	Once daily for 6 weeks	CR 100%	no	-	Moderate erythema	0.5
Guerrieri et Back [89]	1	Radiotherapy	First line	-	60 Gy in 30 fractions	CR 100%	no	-	Moist desquamation	1.0
Moreno-Arias et al. [90]	2	Radiotherapy	First line	-	100 kV, 440 cGy/day, 3 days a week over 3 weeks until a total dose of 3960 cGy was completed	CR 100%	no	-	Hypopigmentation and local atrophy	2.0–3.0
Burrows et al. [91]	5	Radiotherapy	First line	-	4050 cGy rays in nine fractions over 3 weeks	CR 100%	No	-	Moist desquamation	0.5–8.0
Kwan et al. [92]	1	Brachytherapy	First line	-	A total dose of 42 Gy in 14 fractions was delivered at the 5 mm depth from the skin surface over 18 days.	CR 100%	No	-	Wet desquamation, hypopigmentation	0.5
Voigt et al. [27]	1	Systemic chemotherapy	First line	-	Carboplatin administered on day 1 (400 mg/m^2^ intravenously), and calcium folinate on days 1–5 (170 mg/m^2^ intravenously) 1 h before 5-fluorouracil (350 mg/m^2^ intravenously). Six cycles were performed. Treatment cycles were repeated every 4 weeks.	CR 100%	No	-	Thrombocytopenia, necessitating dosage modification	1.0
Marchitelli et al. [93]	10	5% Imiquimod cream	First line	-	cream was applied every other day until clinical lesions disappeared (3 to 6 months)	CR 80%	No	-	Local reaction	1.5
Karam et Dorigo [28]	92	Radiotherapy	First line (41) Adjuvant (51)	Surgical treatment	evaluated patients diagnosed with invasive EMPD using data collected from the SEER program. radiation fields, doses, fractionation schedules and radiation sources vary widely making these reports difficult to compare	-	-	-	-	-
Machado et al. [94]	1	5% Imiquimod cream	First line	-	Three times/week for three months	CR 100%	No	-	-	0.5
Makino et al. [26]	2	Boron neutron capture therapy (BNCT) (experimental form of RT)	First line	-	The tumors were irradiated at the Kyoto University Research Reactor with thermal neutrons of 5 MW for 65 min and 78 min, respectively	CR 100%	No	-	-	1.0
Schmid et al. [95]	1	5% Imiquimod Cream	First line	-	Once daily for 3 months	CR 100%	No	-	-	1.0
Tae Heung et al. [79]	1	Radiotherapy	Adjuvant	Surgical treatment (positive margin) Imiquimod	Imiquimod ointment was applied for 2 months, followed by multiple skin biopsies, which revealed Paget’s disease in all specimens. A total dose of 5040 cGy in 28 fractions was given within a 3-month period	CR 100%	No	-	desquamation of the radiation-exposed skin	1.0
Besa et al. [96]	9	Radiotherapy	First line (4) Adjuvant (5)	Surgical treatment	Doses ranged from 40 to 60 Gy	CR 100%	No	-	Local erythema, swelling, pain, and rectal urgency	1.0
Choi et al. [1]	10	5% Imiquimod cream	Adjuvant	Surgical treatment	Postoperatively, topical imiquimod was applied every other night, 3 times a week, over the margin and adjacent normal skin for a period of 6 months	CR 100% (no recurrence observed)	No	-	-	6.0
van der Linden et al. [97]	10	5% Imiquimod cream	First line	-	Once daily for 10–24 weeks (median 13)	CR 40%, PR 40%, NR 20%	-	Surgical treatment	-	3.3
van der Linden et al. [98]	18	5% Imiquimod Cream	First line	-	-	CR 20%, PR 40% NR 20%	-	-	-	3.0
Wang et al. [99]	13	Surgery (5) ALA-PDT + surgery (8)	Neoadjuvant	-	Four sessions of topical PDT mediated with 20% 5-aminolevulinic acid (ALA-PDT) were applied prior to surgery	CR (40%) CR 63%	Yes 25% 9%	-	Pain during light irradiation	1.0
Chen et al. [100]	16	Photodynamic therapy	Adjuvant	Surgical treatment	Postoperatively, all patients underwent three courses of ALA PDT to the operative site and nearby areas	CR 100%	Recurrence rate 12.5%	Surgical treatment	Infection, lower limb movement disorder	3.0
Zhou et al. [36]	36	A)Wide local excision (control group, 15 patients) B) Wood lamp examination + 5-ALA-PDT (experimental group, 21 patients)	Neoadjuvant	-	Wood’s lamp with 5-ALA PDT defined tumor margins. 5-ALA emulsion was applied beyond the lesion, fluorescence traced, then biopsies done before resection.	CR 100%	Recurrence rate 26.7% 28.6%	-	Local pain during light irradiation	4.0

## 4. Discussion

Biopsy remains the definitive diagnostic procedure for EMPD, yet several non-invasive imaging modalities have emerged as valuable adjunctive tools. These techniques, while not definitive, may facilitate earlier detection, refine treatment planning, and reduce the need for repeated biopsies.

RCM is the most extensively investigated. Multiple studies report strong correlation with histopathology in detecting Paget’s cells and tumor nests in the epidermis [11,28,41]. Characteristic findings consist of large hyporeflective cells with well-defined hyperreflective borders, observed either individually or grouped in small nests within the epidermis. Beyond diagnosis, RCM enables intraoperative margin mapping and postoperative assessment to improve surgical precision. However, false negatives remain an issue [41], emphasizing the need for standardized criteria and validation in larger cohorts.

PDD, although not specific for EMPD, has proven useful for margin delineation. Fluorescence often reveals subclinical spread beyond visible margins, guiding both surgery and PDT [37]. When combined with RCM, PDD appears to improve margin accuracy, reducing overtreatment and preserving tissue while maintaining oncological safety [32].

Dermoscopy may highlight suspicious features, aiding lesion triage for biopsy, but lacks specificity for EMPD [5].

Overall, current evidence supports RCM and PDD as the most reliable non-invasive tools in EMPD, particularly for diagnosis and margin assessment. Dermoscopy and OCT remain investigational, while MRI and PET/CT appear promising for staging and monitoring. These methods should be regarded as complementary to histopathology. Further, large-scale, multicentric prospective studies are needed to validate diagnostic accuracy, standardize imaging markers, and clarify their impact on recurrence and long-term outcomes [5].

The evidence supporting the use of topical imiquimod for EMPD remains fragmentary and largely based on small series or isolated case reports, with only a few larger cohort studies available. This inevitably introduces publication bias and limits the strength of current recommendations. In several studies, imiquimod was used either as a first-line approach or as an adjuvant following surgery, often reflecting the lack of standardized guidelines across different centers [24,25,64].

Despite these limitations, the available data indicate that imiquimod can induce meaningful therapeutic responses. Larger cohorts such as Borella et al. (*n* = 51) reported a CR in 43.1% of patients (median follow-up of 5.5 years) [50], while van der Linden et al. observed CR in 52.2% of 23 cases (median follow-up of 2.6 years) [55]. Similar results were described by Luyten et al. (52.4% CR, 28.6% PR) [71], whereas Green et al. reported higher rates (78% CR, daily application for 14 weeks) [75]. Smaller series and case reports frequently described complete clearance (100% CR) with varied regimens [51,71], but these outcomes must be interpreted cautiously given the sample size. Interestingly, Escolà et al. documented more heterogeneous results, with CR ranging from 0% to 65% across different treatment arms [51], further underlining the variability of real-world data.

Several factors may influence treatment success. Perianal and multifocal disease have been associated with lower likelihood of CR [50], and studies rarely reported lesion size, although larger and more extensive lesions may correlate with worse outcomes. Treatment regimens were inconsistent, ranging from daily application to two or three times weekly, with durations spanning weeks to more than a year. In the largest series, three to four weekly applications over several months were the most frequent schedules and seemed to achieve balanced efficacy and tolerability [7,75]. Extending treatment beyond six months has been suggested to maximize response [71], but higher frequency increased local toxicity without clearly improving clearance [51].

Adverse events were generally mild to moderate in severity and included erythema, burning, erosions, fatigue, and local tenderness [25,75]. Notably, even patients who interrupted treatment early due to intolerance sometimes achieved CR, raising the hypothesis that the local inflammatory reaction, rather than cumulative dosing, may be the critical determinant of efficacy. Recurrence was reported inconsistently across studies, with some series describing relapses occurring within 12–35 months.

Taken together, current findings suggest that imiquimod is a promising non-surgical alternative for selected EMPD patients, particularly when surgery is contraindicated or refused. However, response rates vary widely across studies, follow-up remains limited, and recurrence is not uncommon. Larger prospective trials with standardized dosing schedules are needed to define its real therapeutic potential, optimize treatment duration, and identify patients most likely to benefit.

PDT has been investigated across different clinical contexts, with efficacy ranging from excellent outcomes to more modest responses. Several reports documented CR in all treated patients. Xiang et al. described durable remission lasting up to 41 months with hematoporphyrin-based PDT [53], while Filonenko et al. reported CR at 6 months following chlorin e6 PDT, although relapse occurred at 21 months [45]. Gao et al. documented CR in all 38 patients treated with ALA-PDT in either the adjuvant or first-line setting, but recurrences were noted at 6 and 12 months [24]. Other smaller series confirmed high short-term CR rates, such as Al Youssef et al. with MAL-PDT [68] and Fukui et al. with CO_2_ laser plus PDT [78]. By contrast, more modest efficacy was observed in other cohorts. Rioli et al. reported CR in only 15% of patients, with partial or stable disease in the majority [47], while Fontanelli et al. documented a CR rate of just 3.9%, with most patients achieving only partial responses and multiple relapses during follow-up [48].

Adverse events were common but generally manageable. The most frequent were local pain, erythema, edema, and swelling [24,52]. More severe local reactions such as necrosis, ulceration, and scarring were reported occasionally [45,52]. Additional side effects included photosensitivity, infection, and hyperpigmentation [32,49].

Recurrence rates varied considerably. While some single-case reports described durable remission without relapse [52,68], larger series showed higher recurrence: Gao et al. noted relapses within 12 months despite initial CR [24], Fontanelli et al. reported multiple recurrences during follow-up [48], and Chen et al. and Zhou et al. documented recurrence rates of 12.5% and 26.7%, respectively [36,100].

These findings indicate that PDT can induce high initial response rates, including complete remissions in a proportion of patients. Although high response rates are achievable, maintaining long-term remission remains challenging, as relapse may occur even after initial complete clearance.

Although current evidence supports the diagnostic and therapeutic potential of non-invasive imaging and topical or photodynamic approaches in EMPD, several methodological and practical limitations restrict their clinical applicability. Most available studies are retrospective, single-center, and include small or heterogeneous patient populations, often combining primary and recurrent cases or different anatomic sites. Such variability complicates cross-comparison and may account for the wide range of reported complete response rates. Furthermore, inconsistent endpoints—clinical versus histological clearance—and heterogeneous follow-up durations make it difficult to assess true long-term efficacy and recurrence risk.

From a clinical standpoint, these findings highlight the need for a tailored, multimodal strategy rather than a one-size-fits-all approach. RCM and PDD may be best positioned as adjuncts for preoperative mapping and postoperative surveillance, but they cannot yet replace histopathology as the gold standard. Similarly, imiquimod and PDT should be considered options for carefully selected patients—particularly those unfit for or unwilling to undergo surgery—rather than routine alternatives. Treatment selection should be guided by disease extent, anatomical site, and patient comorbidities, while acknowledging the absence of standardized protocols.

While non-surgical modalities can achieve meaningful responses in selected patients, surgical management remains the cornerstone of EMPD treatment, providing superior local control and lower recurrence rates. Margin-controlled techniques, including MMS, show recurrence rates of about 11%, compared with roughly 37% for WLE [13]. Conservative or limited resections may be appropriate for frail or elderly patients, though recurrence risk is higher. By contrast, topical imiquimod achieves complete responses in 30–54% of cases, but recurrence occurs in up to 35% and local irritation is common [18]. PDT can induce high initial clearance, yet long-term remission is less consistent. Overall, surgery—particularly margin-controlled approaches—should be considered the preferred curative option whenever feasible, while conservative or nonsurgical treatments are best reserved for patients with contraindications or high surgical morbidity.

Future investigations should prioritize prospective, multicentric trials with standardized imaging protocols and uniform response criteria. For imaging, establishing validated diagnostic markers and reproducible interpretation guidelines is crucial to minimizing observer variability and false negatives. For topical and photodynamic therapies, randomized controlled studies comparing regimens, durations, and combination strategies are needed to clarify optimal dosing and maintenance approaches. Long-term follow-up data are also essential to determine whether initial complete responses translate into durable remission.

Finally, translational studies exploring biomarkers of treatment response and predictors of recurrence could refine patient selection and move the field toward personalized therapy. Integration of molecular profiling with advanced imaging may eventually allow more accurate staging, dynamic monitoring, and early detection of relapse—key steps toward improving outcomes in EMPD.

## 5. Single-Center Case Series

As a complement to the findings of our systematic review, we present our single-center experience and case series of EMPD treated with topical imiquimod and monitored longitudinally with RCM.

In our clinical experience, we have treated three patients with penile or scrotal EMPD using topical imiquimod. All patients underwent regular follow-up visits every six weeks and were monitored longitudinally with RCM, during which treatment continuation was decided based on the presence or absence of microscopic disease persistence.

The first case involved a 74-year-old man with recurrent penile EMPD after first-line surgery. He was treated with imiquimod five times per week for six-week cycles, with complete remission achieved after several courses and stable disease-free follow-up at one year.

**Figure 2:**
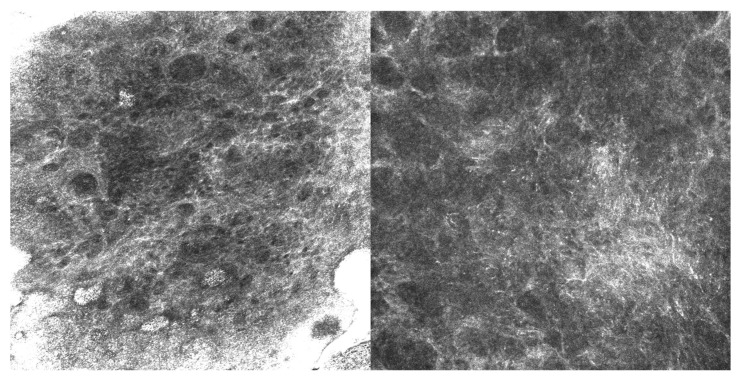
RCM images (500 µm × 500 µm) showing Paget’s cells with clearly defined hyperreflective borders and hyporeflective cytoplasm, larger than the surrounding keratinocytes and scattered within the spinous layer.

The second case, a 63-year-old man with EMPD, also received imiquimod following surgical excision with positive margins. He experienced a microscopic recurrence, which was documented by RCM (Figure 2) and required retreatment; he subsequently remained stable without evidence of disease at one-year follow-up.

The third case concerned a 52-year-old man with EMPD and positive surgical margins. After initiation of imiquimod, complete clinical and microscopic remission was obtained within six months.

## 6. Conclusions

Current knowledge about EMPD management remains limited and not straightforward: the disease is rare, presents differently across patients, and there are no universally accepted therapeutic rules to follow. The literature, mostly made of small case series and retrospective reports, suggests that non-invasive imaging can help improve diagnostic accuracy. Still, this remains within limits, since such methods are not standardized for this condition, and their role is more supportive than definitive in planning treatment or follow-up. On the therapeutic side, topical immunotherapy and PDT have shown encouraging results, especially when surgery is either not possible or would be excessively harmful.

All of this underlines the need for prospective studies able to clarify diagnostic criteria, refine treatment choices, and offer reliable outcome measures. Combining new diagnostic tools with conservative options could open the way to a more individualized and less invasive management of EMPD.

## Data Availability

No new data were created or analyzed in this study. Data sharing is not applicable to this article.

## Abbreviations

The following abbreviations are used in this manuscript:

EMPD	Extramammary Paget’s Disease
RCM	Reflectance Confocal Microscopy
PDT	Photodynamic Therapy
PRISMA	Preferred Reporting Items for Systematic Reviews and Meta-Analyses
WLE	Wide Local Excision
MMS	Mohs Micrographic Surgery
OCT	Optical Coherence Tomography
PET/CT	Positron Emission Tomography/Computed Tomography
MRI	Magnetic Resonance Imaging
ROBINS-I	Risk Of Bias In Non-randomized Studies-of Interventions
CR	Complete Response
PR	Partial Response
SD	Stable Disease
PD	Progressive Disease
ALA	Aminolevulinic Acid
MAL	Methyl Aminolevulinate
HpD	Hematoporphyrin Derivative
BNCT	Boron Neutron Capture Therapy
RT	Radiotherapy
Gy	Gray
MeSH	Medical Subject Headings
DEJ	Dermo-Epidermal Junction
ERBB2-TKI	ERBB2 Tyrosine Kinase Inhibitor
NC	No Change
SEER	Surveillance, Epidemiology, and End Results
